# Synthesis of chitosan nanoparticles (CSNP): effect of CH-CH-TPP ratio on size and stability of NPs

**DOI:** 10.3389/fchem.2024.1469271

**Published:** 2024-11-15

**Authors:** Rosvin E. Des Bouillons-Gamboa, Gabriela Montes de Oca, Jose Roberto Vega Baudrit, Liz Carolina Ríos Duarte, Mary Lopretti, Maite Rentería Urquiza, Juan Miguel Zúñiga-Umaña, Filomena Barreiro, Patricia Vázquez

**Affiliations:** ^1^ School of Biology, Tecnológico de Costa Rica, Cartago, Costa Rica; ^2^ LANOTEC CENAT CONARE, San José, Costa Rica; ^3^ Research Department of Food Engineering and Technology, National University of Asunción, San Lorenzo, Paraguay; ^4^ Department of Biochemistry and Biotechnology, Center for Nuclear Research, Faculty of Sciences, University of the Republic, Montevideo, Uruguay; ^5^ Department of Chemistry, University of Guadalajara, Guadalajara, Mexico; ^6^ Polytechnic Institute of Bragança (IPB), Bragança, Portugal; ^7^ Universidad Nacional de la Plata, La Plata, Argentina

**Keywords:** chitosan, nanoparticles, synthesis, antibiotic resistance, AFM, DLS, TEM

## Abstract

In the face of a pressing global issue-the escalating threat of antibiotic resistance-the development of new antimicrobial agents is urgent. Nanotechnology, with its innovative approach, emerges as a promising solution to enhance the efficacy of these agents and combat the challenge of microbial resistance. Chitosan nanoparticles (CSNPs) stand out in biomedical applications, particularly in the controlled release of antibiotics, with their unique properties such as biocompatibility, stability, biodegradability, non-toxicity, and simple synthesis processes suitable for sensitive molecules. This study synthesized CSNPs using the ionotropic gelation method, with tripolyphosphate (TPP) as the crosslinking agent. Various CS: TPP ratios (6:1, 5:1, 4:1, 3:1, 2:1) were tested, and the resulting nanoparticles were evaluated using dynamic light scattering (DLS). The CS: TPP ratio of 4:1, with an average hydrodynamic diameter (DHP) of (195 ± 10) nm and a zeta potential of (51 ± 1) mV, was identified as the most suitable for further analysis. The characterization of NPs by Transmission Electron Microscope (TEM) and atomic force microscopy (AFM) revealed diameters of (65 ± 14) nm and (102 ± 18) nm, respectively. Notably, CSNPs exhibited significant aggregation during centrifugation and lyophilization, leading to diameter increases of up to 285% as measured by AFM. The antibacterial activity of CSNPs against *Staphylococcus aureus* and *Escherichia coli* was assessed using the resazurin assay. It was found that CSNPs not subjected to centrifugation, freezing, and lyophilization retained their antimicrobial activity. In contrast, those that underwent these processes lost their efficacy, likely due to aggregation and destabilization of the system. This study presents a straightforward and effective protocol for encapsulating sensitive active agents and synthesizing chitosan nanoparticles, a potential system with significant implications in the fight against antibiotic resistance.

## 1 Introduction

The definition of a nanometer as one trillionth of a meter (10^−9^ m) and the range of the nanometer scale, which extends from the atomic level (0.2 nm) to 100 nm, was established in the 2004 report by the United Kingdom Royal Society and Royal Academy of Engineering. In this range, materials exhibit improved or distinct properties compared to their larger counterparts, mainly due to increased relative surface area and significant quantum effects (The Royal Society and The Royal Academy of Engineering, 2004). Nanotechnology, defined in 2005 by Kelsall, Hamley, and Geoghegan as the science of designing, fabricating, and using nanostructured systems, is classified into two main manufacturing approaches: the ‘bottom-up’ and the ‘top-down.’ The ‘bottom-up’ approach, first described by Richard Feynman (in 1960), involves building structures atom by atom or molecule by molecule through chemical methods, self-assembly, and positional assembly. On the other hand, the top-down approach focuses on the miniaturization of larger structures (The Royal Society & The Royal Academy of Engineering, 2004; Tripathi et al., 2017).

In the field of pharmaceutical sciences, therapeutic nanotechnology, as an emerging field, has revolutionized modern medicine, as nanoparticles (NPs) in ranges of 10–100 nm are used as vectors for the protection and controlled release of therapeutic molecules, improving their biodistribution and effectiveness (Kosta et al., 2012; Watkins et al., 2015; Saeedi et al., 2019). In particular, chitosan nanoparticles (CSNPs) have been noted for their physicochemical characteristics, including biocompatibility, mucoadhesivity, biodegradability, solubility, non-toxicity, and ease of synthesis, which has encouraged their use in the controlled release of antibiotics (Duceppe and Tabrizian, 2010; Shukla et al., 2013; Kamat et al., 2016; Safdar et al., 2019; Xu et al., 2022; Qi et al., 2004).

In addition, chitin, the second most abundant polymer in nature and the main component of arthropods’ exoskeleton, gives rise to chitosan through deacetylation. Chitosan is distinguished by its cellulose-like structure and its primary amino group, which gives it unique properties, making it a highly useful polymer for the controlled release of drugs (Puvvada et al., 2012; Shyh, 2013; Saeedi et al., 2022). Chitosan sheets and covers containing natural extracts show excellent antimicrobial and antioxidant properties, being an alternative–as a biodegradable plastic–for food packaging (Muñoz-Tébar et al., 2023; Barik et al., 2024).

On the other hand, within polymeric NPs, CSNPs can be classified as nanocapsules or nanospheres, depending on the distribution of the drug in their structure. The ability of CSNPs to adsorb or absorb active ingredients, such as antibiotics, is crucial in the context of growing resistance to antimicrobial agents, a significant problem in human health and medicine (Blair et al., 2015; Kandile et al., 2018; Wang et al., 2011; Li et al., 2018; Kalantari et al., 2019; Lawson, 2023), including cancer therapies (Rostami, 2020; Rasul et al., 2020; Mushtaq et al., 2021) and diabetes management (Bhardwaj et al., 2020).

Various methods have been used to synthesize CSNPs (Li et al., 2018; Aibani et al., 2021; Bhoopathy et al., 2021), including ionotropic gelation. This method stands out for its simplicity and the friendly conditions under which it is carried out, avoiding the use of organic solvents and extreme conditions (Agnihotri et al., 2004; Duceppe and Tabrizian, 2010; Santo et al., 2021; Ebhodaghe, 2022).

Finally, the characterization of the resulting NPs is essential to defining their properties. Techniques such as Dynamic Light Scattering (DLS), Transmission Electron Microscope (TEM), atomic force microscopy (AFM), and Fourier Transform Infrared Spectroscopy (FTIR) are essential to obtain accurate information about the size, surface area and morphology of NPs (Joshi et al., 2008; Lin et al., 2014).

## 2 Materials and methods

### 2.1 Synthesis and lyophilization of chitosan nanoparticles

Chitosan (CS) (molecular mass 100–300 kDa; DDA: 75%–85%) was dissolved in 1% acetic acid to obtain a CS solution of 2 mg/mL (Gan and Wang, 2007; Koukaras et al., 2012). A tripolyphosphate (TPP) solution was added to this solution at different concentrations to establish various CS/TPP mass/mass ratios (1:1 to 6:1) (Koukaras et al., 2012). TPP was added at a rate of 1 mL/min, under constant stirring at 700 rpm, and stirred for 45 min after dripping was completed (Wu et al., 2005). Finally, different CS/TPP ratios were evaluated to determine the optimal one by analyzing Average Hydrodynamic Diameters (DHP) and Zeta Potential values using Dynamic Light Scattering (DLS). Once the synthesis was complete, the resulting solution was placed in a 50 mL conical tube, previously weighed, and centrifuged for 45 min at 10,000 rpm. Once centrifugation was complete, the supernatant was discarded, and the precipitate was frozen at −20°C, followed by freeze-drying (Cover et al., 2012). The characterization of the CsNPs was performed by Dynamic Light Scattering (DLS), Transmission Electron Microscope (TEM), Atomic Force Microscopy (AFM), and Fourier Transform Infrared Spectroscopy (FTIR).

### 2.2 Determination of antibacterial activity

For the culture of microorganisms, the bacteria (*Escherichia coli* and *Staphylococcus aureus*) -which were cryopreserved-were taken and thawed directly in the Eppendorf tubes, then they were scratched on plates with Müeller Hinton Agar (MH) and allowed to grow for 24 h at 37°C. Subsequently, a sufficient bacterium was taken to be inoculated in ∼15 mL of MH 1X medium and left to incubate at 37°C, 120 rpm, for 2 h. After the time had elapsed, it was centrifuged at 3500 rpm, 10 min at 21°C (Thermo scientific/Sorvall ST16R). The supernatant was discarded, and the pellet resuspended in a saline solution of 0.85% (m/v).

To determine the cell concentration to be used, dilutions were made with 0.85% saline solution (m/v), according to the equation obtained in calibration curves (Lindqvist, 2006; Mira et al., 2022). UV/Visible absorbance spectrophotometer measurements were made to achieve the ideal absorbance for each bacterium (0.055 and 0.039 for *E. coli and S. aureus*, respectively). With the RStudio v1.0.44 program (MA, United States), the optical density correlations for each bacterium were run, where the volume of bacteria to be inoculated was predicted in order to be able to add in total for each well 5 × 10^4^, with the least possible error.

To determine the minimum inhibitory concentration, with the calculated volume, 30 μL of MH 3.3X medium, 10 μL of 0.01% resazurin (m/v), and varying amounts of sterile water and bacteria were added to each of the wells of a plate of 96 wells according to the optical density obtained. In addition, increasing amounts of nanoparticles (5–27.5 μL in 2.5 μL increments) were added per column to have a final volume per well of 100 μL. The concentration of nanoparticles per well and the aggregate volume are detailed in Tables 1–3.

**TABLE 1:** Concentration gradient for treatment with lyophilized and filter-filtered CSNPs from 0.22 μm to a concentration of 5 μg/mL, with respective volumes. They occur for both *S. aureus* and *E. coli.*

Well	μg/mL	μL to take CsNPs	MHB 3.3x	Resazurin	Bacteria	μL of H2O to add	Total Vol μL
1	22	27.5	30	10	5.3	27.2	100.0
2	20	25.0	30	10	5.3	29.7	100.0
3	18	22.5	30	10	5.3	32.2	100.0
4	16	20.0	30	10	5.3	34.7	100.0
5	14	17.5	30	10	5.3	37.2	100.0
6	12	15.0	30	10	5.3	39.7	100.0
7	10	12.5	30	10	5.3	42.2	100.0
8	8	10.0	30	10	5.3	44.7	100.0
9	6	7.5	30	10	5.3	47.2	100.0
10	4	5.0	30	10	5.3	49.7	100.0
11	0	0	30	10	5.3	50.0	95.3
12	0	0	30	10	5.3	50.0	95.3

**TABLE 2:** Concentration gradient for treatment with lyophilized and filter-filtered stock solution CSNPs from 0.22 μm to a concentration of 80 μg/mL, with respective volumes. They occur for both *S. aureus* and *E. coli.*

Well	μg/mL	μL to take CsNPs	MHB 3.3x	Resazurin	Bacteria	μL of H2O to add	Total Vol μL
1	1.38	27.5	30	10	5.5	27.0	100.0
2	1.25	25.0	30	10	5.5	29.5	100.0
3	1.13	22.5	30	10	5.5	32.0	100.0
4	1	20.0	30	10	5.5	34.5	100.0
5	0.88	17.5	30	10	5.5	37.0	100.0
6	0.75	15.0	30	10	5.5	39.5	100.0
7	0.63	12.5	30	10	5.5	42.0	100.0
8	0.5	10.0	30	10	5.5	44.5	100.0
9	0.38	7.5	30	10	5.5	47.0	100.0
10	0.25	5.0	30	10	5.5	49.5	100.0
11	0	0	30	10	5.5	50.0	95.5
12	0	0	30	10	5.5	50.0	95.5

**TABLE 3:** Concentration gradient for treatment with filter-filtered CS stock solution of 0.22 μm at a concentration of 2 mg/mL, with respective volumes.

Well	μg/mL	μL to take CsNPs	MHB 3.3x	Resazurin	Bacteria	μL of H2O to add	Total Vol μL
1	550	27.5	30	10	5.2	27.3	100.0
2	500	25.0	30	10	5.2	29.8	100.0
3	450	22.5	30	10	5.2	32.3	100.0
4	400	20.0	30	10	5.2	34.8	100.0
5	350	17.5	30	10	5.2	37.3	100.0
6	300	15.0	30	10	5.2	39.8	100.0
7	250	12.5	30	10	5.2	42.3	100.0
8	200	10.0	30	10	5.2	44.8	100.0
9	150	7.5	30	10	5.2	47.3	100.0
10	100	5.0	30	10	5.2	49.8	100.0
11	0	0	30	10	5.2	50.0	95.2
12	0	0	30	10	5.2	50.0	95.2

Supplementary Figure 1 shows the organization diagram of the wells in determining the MIC, where rows A and H of the multi-well plate constitute the sterility controls, and columns 11 and 12 correspond to the bacterial growth controls. In columns 1–10 and B-G, the MIC test was performed for each treatment and bacteria. Once finished, the edges were covered with parafilm to prevent dehydration of the wells and incubated for 24 h at 37°C. MIC was determined by the colorimetric change of resazurin, where pink indicates cell viability and blue purple demonstrates bacterial inhibition.

## 3 Results and Discussion

**FIGURE 1:**
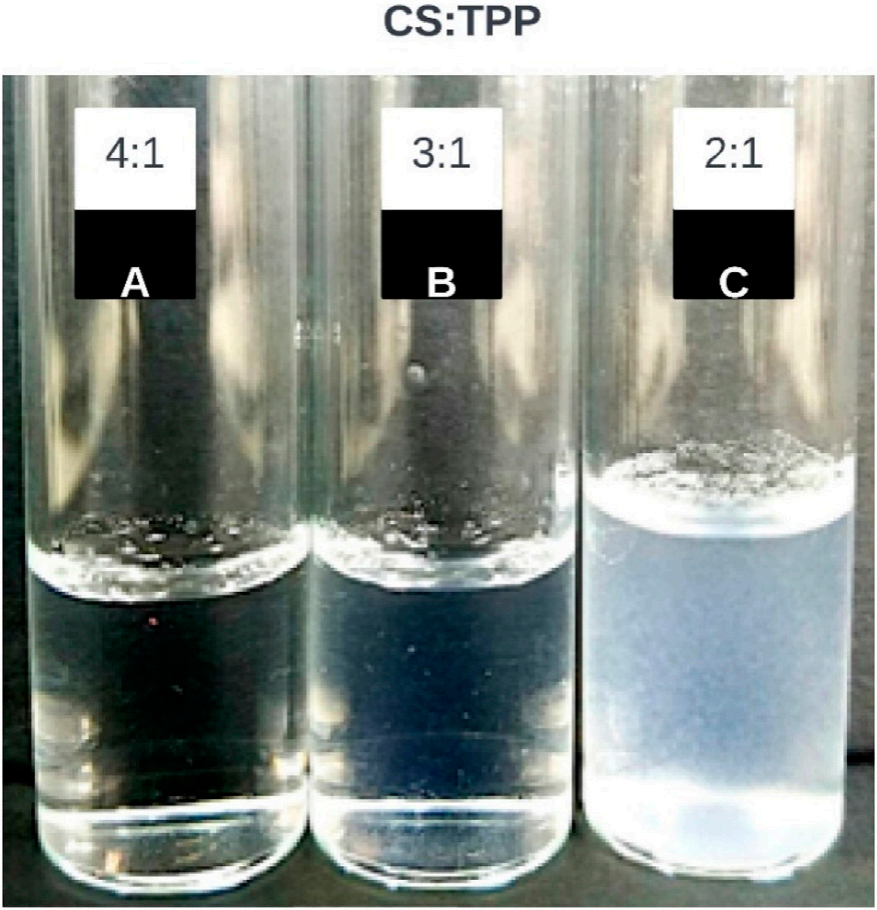
Dissolutions resulting from synthesis to decreasing CSTPP ratios. The concentration of CS was maintained at 2 mg/mL, and the concentration of TPP for each ratio was varied: 2A is (4:1), 2B is (3:1), and finally, 2C is (2:1).

To determine the CS: TPP ratio resulting in flocculation and precipitation of chitosan nanoparticles (CSNPs), five syntheses were performed with ratios ranging from 6:1 to 2:1. According to the results (Figure 1), it is observed that synthesis solutions with 6:1 and 4:1 ratios (2A and 2B, respectively) result in opalescent and clear suspensions. However, for the 2:1 (2C) ratio, flocculation/precipitation is evident, indicating noticeable turbidity. This difference could be attributed to the neutralization of the surface charges of the CSNPs by the excess of TPP, the polyanion used, which also significantly reduces the Zeta potential and leads to the aggregation and precipitation of the NPs, resulting in marked turbidity (Abbas, 2010; Käuper and Forrest, 2006). In contrast, for the 4:1 and 3:1 ratios, the CS: TPP ratio is low enough to maintain a colloidal system of CSNPs without reaching such low ZetaPot values as to cause aggregation and precipitation (Gan et al., 2005). Liu and Gao (2009) have pointed out that the formation of CSNPs is only possible in specific concentrations of CS and TPP. After observing that the CS: TPP ratio of 2:1 generated aggregation in the system, the hydrodynamic diameter (DH) of chitosan nanoparticles (CSNPs) for ratios from 6:1 to 3:1 was measured using Dynamic Light Scattering (DLS). Multiple syntheses were carried out for these four relationships, selecting the three best repetitions for representation in Table 4.

**TABLE 4:** Distributions of Average Hydrodynamic Diameter (DHP) and Potential Zeta (ZetaPot) determined by DLS (average values of 3 samplings of 10 repetitions each, of 5 sweeps each, of 10 s each; the ZetaPot presents the averages of 3 samplings of 5 repetitions each). Values for DHP and ZetaPot with standard error are presented.

CS	DHP [d (nm)]	PDI	ZetaPot (mV)
6:01	202 ± 10	0.563	61 ± 2
5:01	205 ± 12	0.558	53 ± 1
4:01	195 ± 8	0.528	51 ± 1
3:01	178 ± 18	0.472	45 ± 4

The goal was to achieve a nanoparticle system with the minor hydrodynamic diameter possible while maintaining high enough Zeta potential values (ZetaPot) to prevent aggregation and ensure system stability. In addition, we sought to minimize the standard error and polydispersity index (PDI) of the nanoparticles.

Chitosan nanoparticles (CSNPs), regardless of CS: TPP ratio, exhibited mean Zeta potential (ZetaPot) values greater than +30 mV. The Average Hydrodynamic Diameters (DHP) ranged from 178 to 205 nm, varying according to the specific CS: TPP ratio. Specifically, the 5:1 ratio had the highest DHP values (205 ± 12 nm), a polydispersity index (PDI) of 0.558, and a ZetaPot of (53 ± 1) mV. Although the 3:1 ratio showed the lowest values of DHP (178.00 nm), ZetaPot (45.40 mV), and PDI (0.472), the standard errors (ES) associated with these values of DHP and ZetaPot were the highest, 18.30 and 4.35 respectively. In contrast, the 4:1 ratio resulted in a DHP of 194.57 nm with the lowest ES (8.23) of the three treatments evaluated and an average ZetaPot of 51.27 mV with the second lowest ES value (1.37).

**FIGURE 2:**
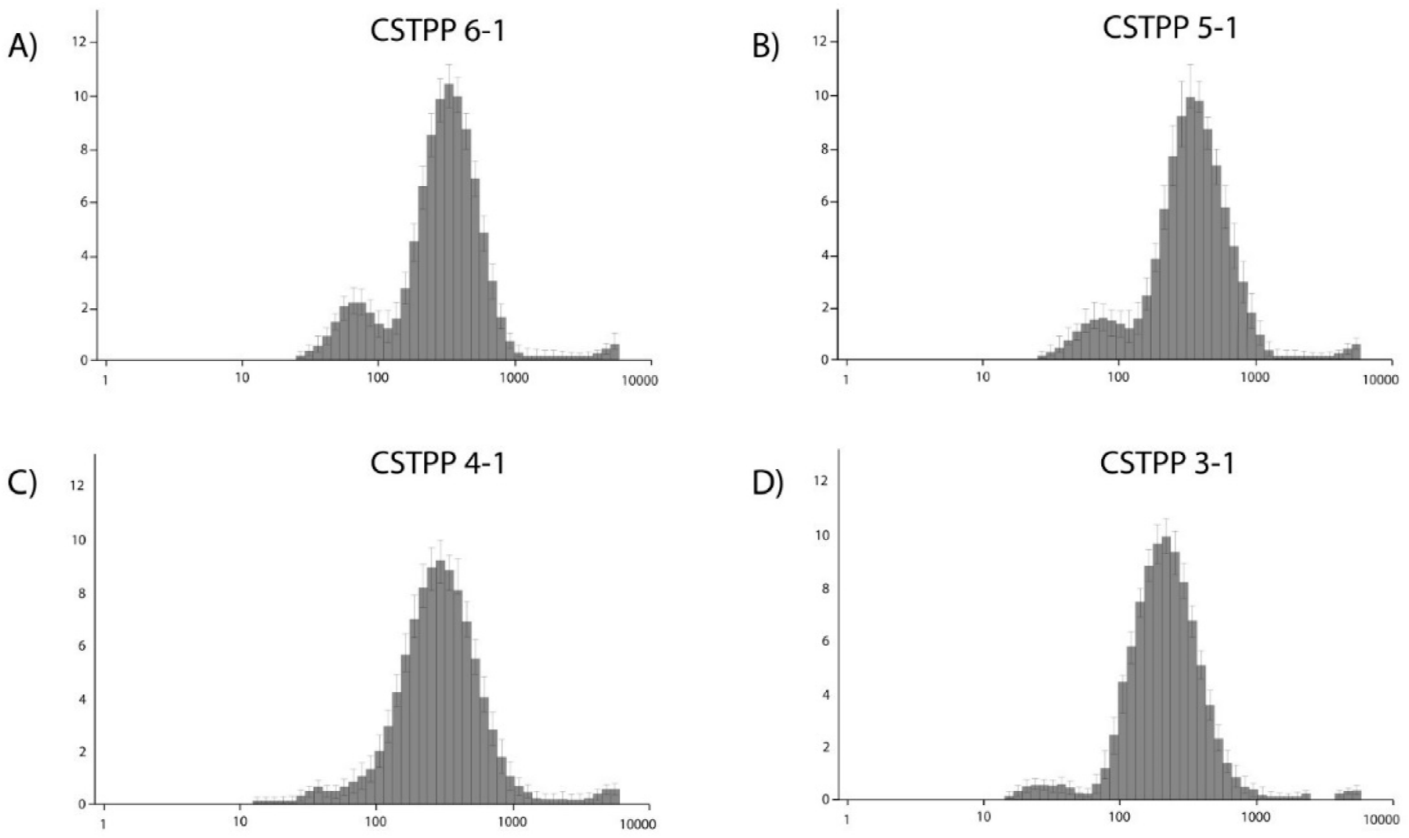
**(A–D)** Graphs of DH distribution of CSNPs by intensity. The following graphs are representative of the 3 DH samplings obtained by DLS. Distribution percentages were obtained directly from the Zetasizer-Malvern Instruments v7.03 program.

Figure 2A illustrates the size distributions for the 6:1 CS: TPP ratio. Here, the main group, with an average size of 203 nm, makes up 84.10% of the distribution. In addition, there is a subgroup with an average size of 60 nm, representing 11.8% of the distribution. Figure 2B, corresponding to the 5:1 ratio, shows a main group with average sizes of 205 nm, covering 83.10% of the distribution, and a subgroup with average sizes of 75 nm, representing 16.90%. In Figure 2C, for the 4:1 ratio, the major group with average sizes of 195 nm accounts for 81.90% of the distribution, while a subgroup of about 50 nm comprises 18.30%. Finally, in Figure 2D, which shows the distribution for the 3:1 ratio, the leading group with average sizes of 178.00 nm constitutes 87.50% of the distribution, and a subgroup with sizes close to 40 nm accounts for 10.40%.

The Average Hydrodynamic Diameter (DHP) results obtained by Dynamic Light Scattering (DLS) indicate a decreasing trend of DH as the CS: TPP ratio decreases. As shown in Table 4, the 6:1 ratio resulted in DHPs of (202 ± 10) nm, while the lowest ratio, 3:1, produced DHPs of (178 ± 180) nm. These findings are consistent with those of Liu and Gao (2009), who found that the higher the concentrations of TPP, the smaller the size of the particles. The decrease in DH could be due to a greater degree of crosslinking facilitated by the availability of TPP, resulting in a more compact structure of NPs (Duceppe and Tabrizian, 2010). Conversely, at lower concentrations of TPP, NPs with lower structural densities are formed due to the increase in distances between interaction and crossover sites, increasing particle size (Koukaras et al., 2012). Tsai et al. (2004) suggest that this CS: TPP-specific ratio leads to the highest crosslinking efficiency, while higher ratios result in less efficient crosslinking, influencing the increase in particle size.

The ZetaPot values obtained were all positive, consistent with what is expected for CSNPs synthesized by this method (Oudih et al., 2023). It was also observed that the higher the CS: TPP ratios, the more ZetaPot values tend to increase (Kouchak et al., 2012), attributable to a higher density of protonated amino groups (Yang and Hon, 2009). A ZetaPot of ± 30 mV generally indicates stability in the particles, while lower values suggest instability and a tendency to aggregation (Sapsford et al., 2011).

Although the results of this study are consistent with other reports, variations are observed in the optimal CS: TPP ratio, as well as in particle sizes, ZetaPot, and polydispersity indices, influenced by factors such as the degree of deacetylation (DDA) and molecular mass of the CS (Wang et al., 2011). Differences in DDA and molecular masses of CS used in different studies may explain the variations in size and distributions observed in DLS measurements. Abbas (2010) found that CS chains with decreasing DDA result in larger NPs, while Goycoolea et al. (2004) and Csaba et al. (2009) noted a significant increase in the size of NPs with higher molecular mass CSs. These effects of molecular mass and DDA on the size of NPs may be due to changes in viscosity and the ability of low-molecular-mass CS to form smaller structures.

**FIGURE 3:**
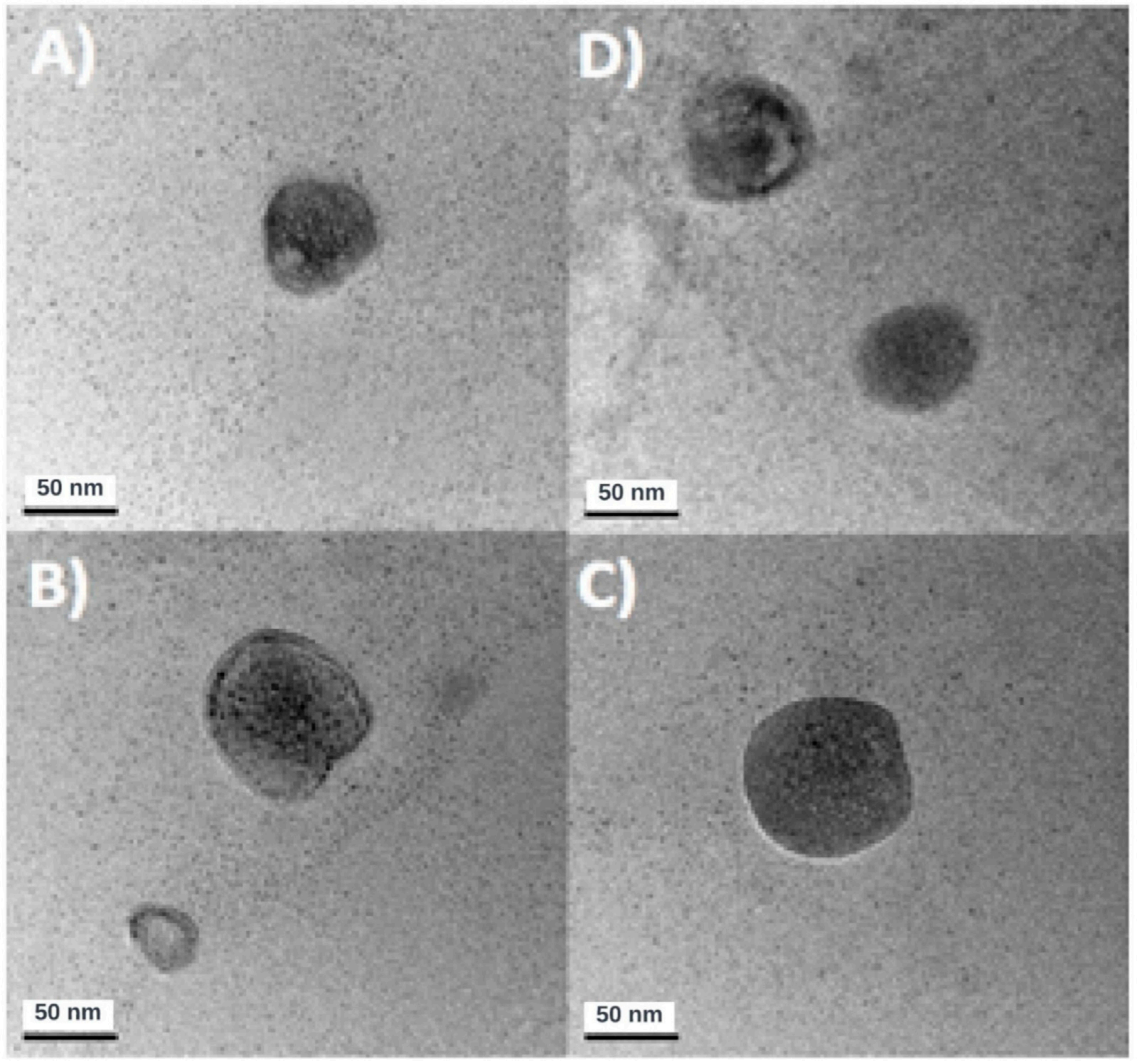
**(A–D)** Images for CSNPs 4:1 obtained by TEM.

Likewise, a higher molecular mass is associated with a higher polydispersity index (PDI) for the resulting particle sizes (Rampino et al., 2013). Considering the wide range of molecular mass (100–300 kDa) and the DDA (75%–85%) of the CS used in this study, the PDI values obtained (0.472–0.563) can be explained (Table 4). In addition, this wide range of molecular mass may explain the observed size distributions (Figure 3), with a larger main population and a smaller subpopulation, in agreement with Tsai et al. (2004).

The CS:TPP ratio 4:1 was selected for subsequent tests because it had adequate DHP, ZetaPot, and dispersion values for the system’s target. Although the 3:1 ratio offered lower values of DHP and PoI, the standard error values and ZetaPot were not considered optimal for NPs as a controlled drug release system, similar to what was reported by Calvo et al. (1997), who also chose a different ratio than the one that produced the lowest DHP values.

**FIGURE 4:**
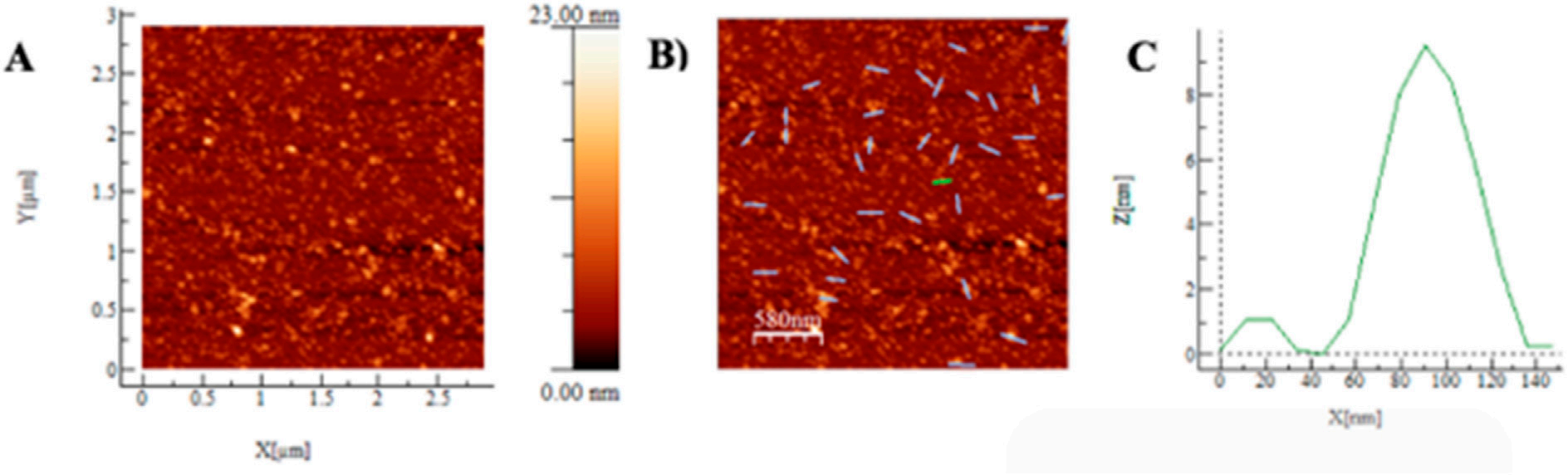
**(A–C)** AFM results of non-lyophilized 4:1 CSNPs. In 5A, the graph obtained by AFM in intermittent mode is presented, with the X, Y, and Z axes. In 5B, the 32 zones that were taken to determine the diameter and average heights are presented with lines.

Figure 3 shows 4 TEM images representative of the sampling of NPs in the 4:1 CSTPP ratio. The nanoparticles (NPs) obtained had an average diameter of (65 ± 14) nm, calculated after measuring the diameter of 13 NPs. Four representative images of CSNPs with a CS: TPP ratio of 4:1, diluted 1:50, are included, where each image shows a 50 nm scale in its lower left corner. The data are presented with their respective standard deviation. There is a notable difference between these results and those obtained by Dynamic Light Scattering (DLS), where the Average Hydrodynamic Diameter (DHP) was (195 ± 9) nm, as detailed in Table 4. This discrepancy can be explained by the differences inherent in each characterization method. The values obtained by DLS are usually higher, with differences in measurement between DLS and Transmission Electron Microscope (TEM) that can vary between 2 and 4 times (Mahl et al., 2011). This is because DLS measures the hydrodynamic radius, considering the electrical double layer that includes the Stern and diffuse outer layers (Clogston and Patri, 2011). In addition, in DLS, CSNPs are measured in a hydrated state within the solution. On the other hand, in TEM characterization, NPs are subjected to high vacuum and desiccation conditions, resulting in measurements of NPs in their least hydrated state, thus presenting considerably smaller diameters. This behavior has been described by Mahl et al. (2011) and Lee et al. (2014) (Table 5). For a third reference, the same NPs (CSTPP 4:1) were characterized by AFM. Images and data on the diameter and height of the NPs were obtained (Figure 4).

**TABLE 5:** Differences in NP size due to different characterization methods (DLS vs. TEM).

Author	Size [d (nm)]		Material
	DLS	TEM	
Lee et al. (2014)	128.5 ± 5.7	86	Silica
	109 ± 3.1	68	
Mahl et al. (2011)	42	15 ± 1.5	Au
Wu et al. (2005)	∼120	20–80 nm	CSTPP

In the study, 32 zones were analyzed, obtaining an average diameter and height of (102 ± 18) nm and (10 ± 3) nm, respectively (5C). A representative profile is shown in Figure 4C, marked with a green line. In this profile, a 100 nm main particle with a height of 9.5 nm and a 35 nm secondary particle with a height of 1.0 nm are observed. The data is presented with its respective standard error.

**FIGURE 5:**
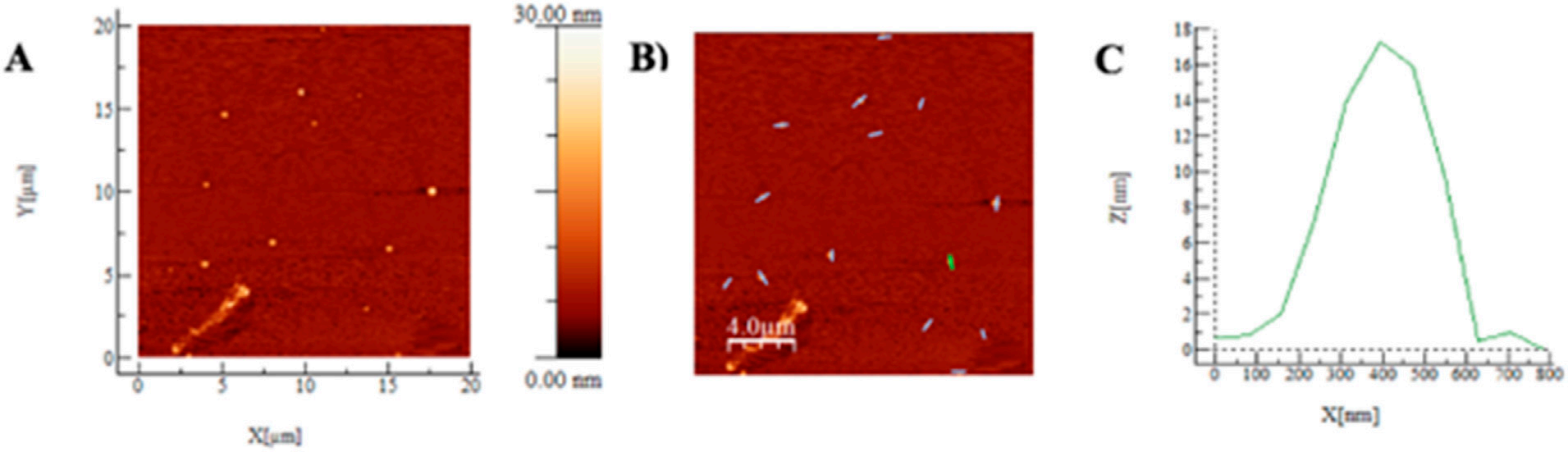
**(A–C)** AFM results of lyophilized 4:1 CSNPs. In 5A, the graph obtained by AFM in intermittent mode is presented, with the X, Y, and Z axes. In 5B, the 13 zones that were taken to determine the diameter and average heights are presented with lines.

Considering the Average Hydrodynamic Diameter (DHP) values of (195 ± 93) nm obtained by DLS and the TEM values of (65 ± 14) nm, where the nanoparticles (NPs) are in their minimum hydration state, an estimate of the size of CS: TPP CSNPs 4:1 is achieved. Atomic Force Microscopy (AFM) measurements indicate that the samples are in a state of semi-hydration, so it was expected to obtain diameter measurements between TEM and DLS values (Vůjtek et al., 2007). The results were an average diameter of (102 ± 18) nm and an average height of (10 ± 3) nm. However, it is essential to consider that in AFM, lateral dimensions may be affected by sample-tip convolution, which could cause diameter oversizing (Vůjtek et al., 2007; Sedin and Rowlen, 2001). In addition, interactions between CSNPs, with their hydrogel nature, and the hydrophilic substrate can influence the observed dimensions, promoting a structure with a larger diameter and a smaller height, as illustrated in Figure 5 (Zhao et al., 2015). This correlates with clusters of varying sizes observed in DLS and TEM results.

Usually, the freeze-drying method is used to store and preserve nanoparticulate formulations. It has been reported that CSNPs obtained by the ionotropic gelation method tend to lose integrity if they are kept in solution (López-León et al., 2005). For this reason, CSTPP 4:1 CSNPs that underwent this freeze-drying process and, after being resuspended, were characterized by AFM (Figure 5).

In the analysis of 14 sampled areas, an average diameter and height of (558 ± 179) nm and (13 ± 5) nm were obtained, respectively. A representative profile, marked with a green line, is shown in Figure 5C, where a 450 nm main particle with a height of 18 nm and a secondary particle of 150 nm with a height of 1.0 nm are highlighted. These data are presented with their respective standard deviation. When comparing the Atomic Force Microscopy (AFM) data in Figures 4, 5, a significant increase in diameter is observed, going from (102 ± 18) nm to (558 ± 179) nm. This increase is consistent with the findings of Abbas (2010), who reported that when lyophilizing CS-Dextran nanoparticles (NPs), they increased from (137 ± 4) nm to (314 ± 23) nm, representing an increase of 129.2%. This author also reported increases of 142.8% for CS: TPP NPs. In addition, he highlighted the importance of cryoprotectants in protecting NPs against aggregation and other undesirable changes during lyophilization (Table 6). It is relevant to note that in this study, a cryoprotectant was not used during the lyophilization of chitosan nanoparticles (CSNPs), which could be a determining factor in the aggregation observed in the particles, according to the data obtained by Atomic Force Microscopy (AFM) presented in Figure 5. In addition, it is essential to consider the findings of López-León et al. (2005), who reported that freezing a solution of CSNPs at −10°C and thawing it after 1 month of storage resulted in complete system destabilization. It was even pointed out that the NPs became utterly unusable for any application after this freezing and thawing process. As a result, subsequent research has chosen to use trehalose as a cryoprotectant for NPs.

**TABLE 6:** Effect of different cryopreservatives on size variation and ZetaPot after a freeze-drying process.

Author (Year)	NPs	Variation (%)		Cryopreservative
		Size	ZetaPot	
Abbas, (2010)	CS-DS	129.2	42.7 ± 6.8	-
		6.6	4.1 ± 2	Sucrose
		36.9 ± 8.2	-	Mannitol
		20.1 ± 3.7	-	Sorbitol

CS-DS,Chitosan-Dextran NPs.

**FIGURE 6:**
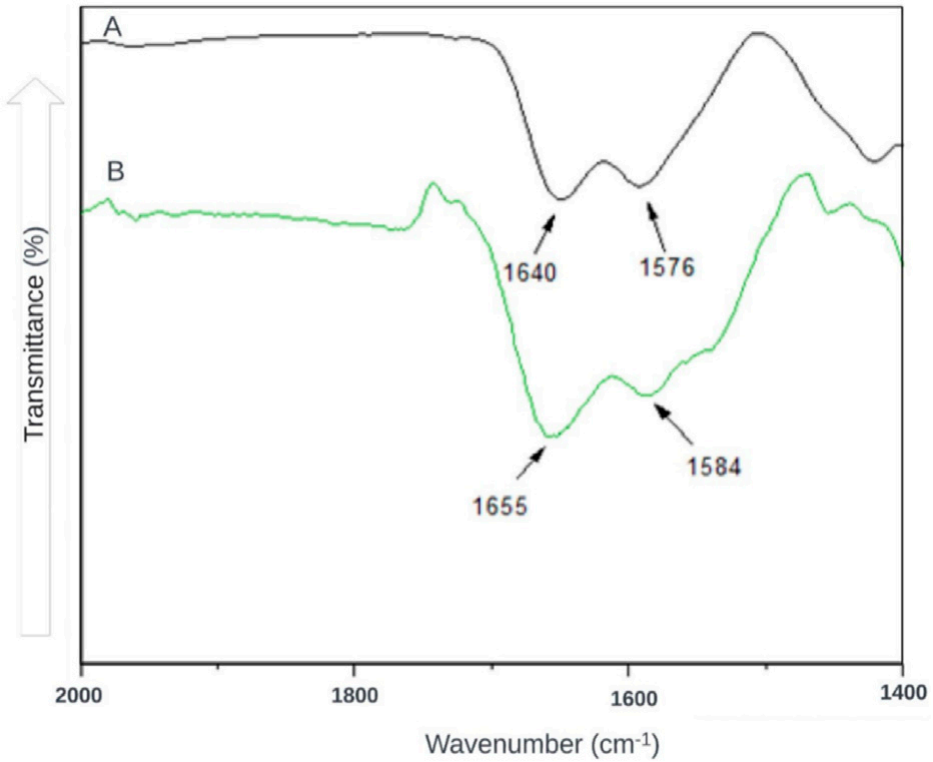
In Fourier Transform Infrared Spectroscopy (FTIR), the spectrum of CSNPs is represented by the black line **(A)**, while the green line represents that of CS **(B)**.

The FTIR characterization method (Figure 6) was also used to identify possible interactions between CS and TPP at the molecular level. Lyophilized NPs were compared with the CS used for synthesis. The results obtained agree with Xu and Du (2003) and Rampino et al. (2013), where the band at 1,655 cm^−1^ and 1,584 cm^−1^ in 8B and 1,640 cm^−1^ and 1,576 cm^−1^ in 8A are attributed to the interaction between phosphate (TPP) and ammonium (CS) ions.

**FIGURE 7:**
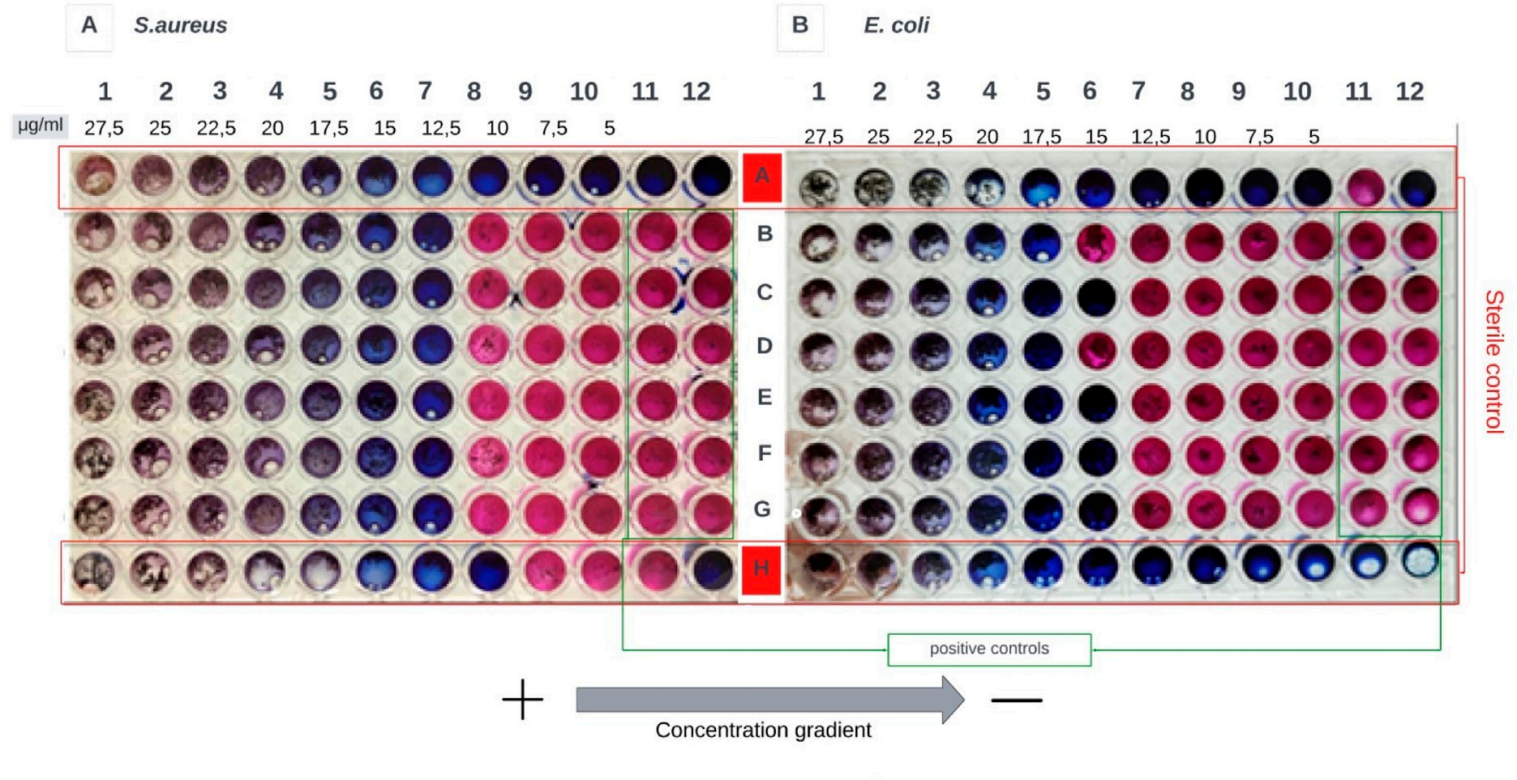
The result obtained for the test with CSNPs CSTPP ratio 4:1 in their synthesis solution. **(A)** presents the results for *S. aureus* and **(B)** the results for *E. coli.* Both E*. coli* and *S. aureus* were performed in duplicate, and inhibition was obtained in the same column (n = 3) for both.

To determine the Minimum Inhibitory Concentration (MIC), a first approach was made to determine the antimicrobial activity of the resulting NPs in the synthesis solution. Figure 7 shows the image obtained for the test to determine the antimicrobial activity of CSNPs on the bacterium *Escherichia coli (E. coli)* and *Staphylococcus aureus (S. aureus).* All results presented are for CSNPs CSTPP ratio 4:1.

In Figure 7A, growth inhibition occurs in column 6 for *Staphylococcus aureus*. On the other hand, in Figure 7B, the inhibitory effect in *Escherichia coli* is evidenced in column 7. Three models have been proposed to explain the interaction between positively charged chitosan nanoparticles (CSNPs) and the negatively charged bacterial membrane. These interactions are primarily mediated by electrostatic forces, possibly competing with Ca^2+^ ions for electronegative sites in the cell membrane. This electrostatic interaction induces changes in the permeability of the cell wall, causing internal osmotic imbalances that affect the growth of microorganisms (García and Pérez, 2002). In addition, this interaction can cause the hydrolysis of peptidoglycans in the cell wall, leading to the release of intracellular electrolytes and other low molecular mass components such as proteins, nucleic acids, glucose, and lactate dehydrogenase (Islam et al., 2011).

These mechanisms may explain the results shown in Figure 7, where growth inhibition is observed at higher concentrations of CSNPs, similar to what is reported in Figure 10A since the mechanisms of action between CS and CSNPs are similar. After obtaining preliminary results on the activity of NPs in solution, additional tests were carried out to evaluate the minimum inhibitory concentration (MIC) of NPs, not in their synthesis solution, but after centrifugation, freezing, and lyophilization. This approach is because lyophilization is a commonly employed method both to determine the concentration of NPs resulting from synthesis and for their storage since keeping PNs in solution for prolonged periods can result in destabilization or even total degradation of the system (López-León et al., 2005).

**FIGURE 8:**
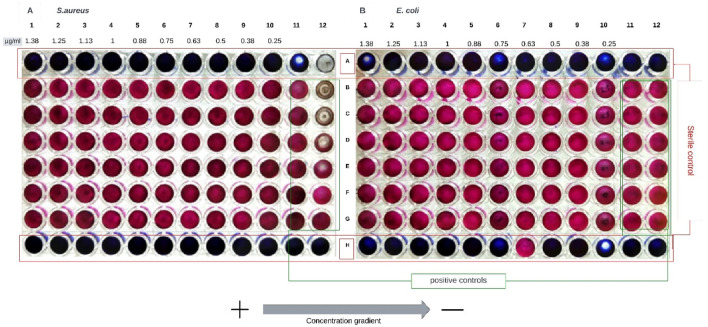
The result obtained for the MIC of the NPs resulting from the centrifugation, freezing, and lyophilization process is shown. **(A)** presents the results for *S. aureus* and **(B)** the results for *E. coli*. A 5 μg/mL stock solution was used for the assay, and then a concentration gradient from 1.38 μg/mL to 0.25 μg/mL was obtained.

**FIGURE 9:**
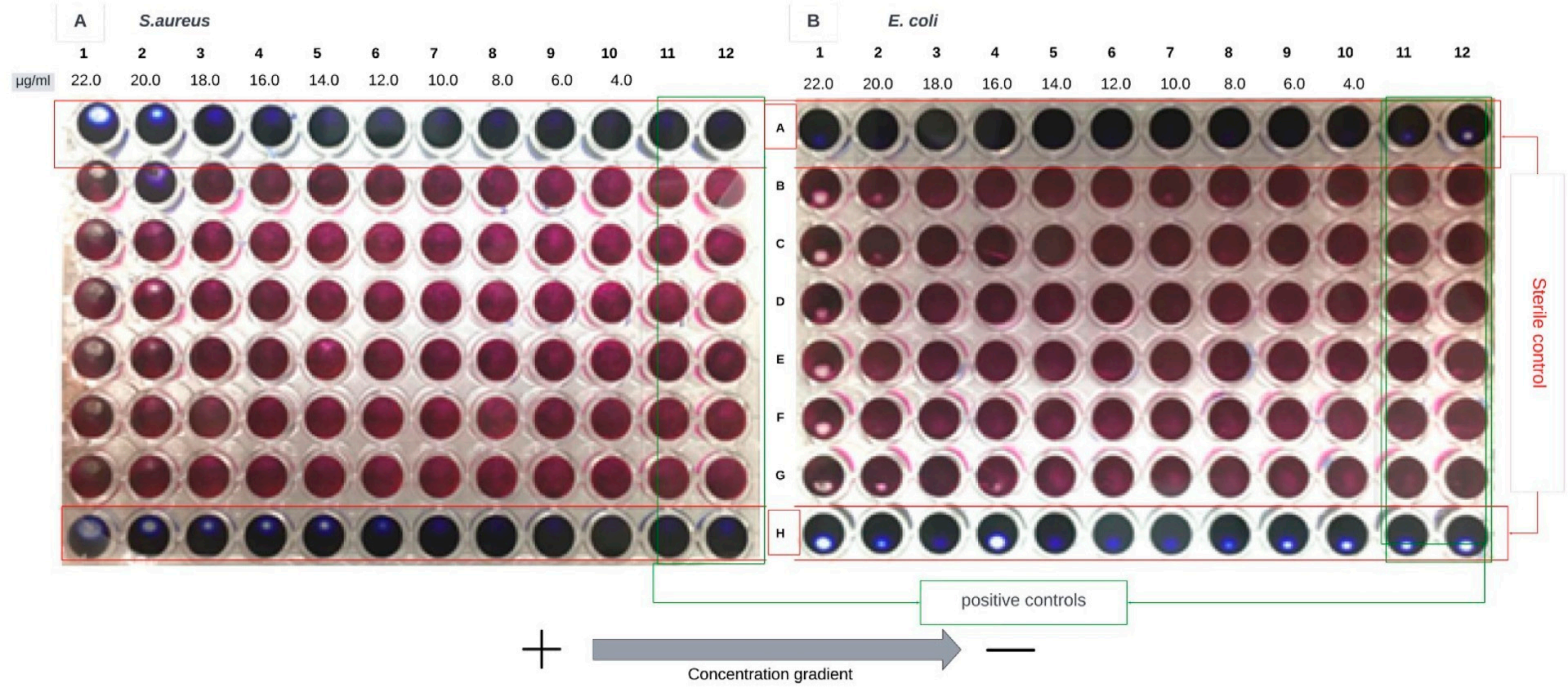
The result obtained for the MIC of the NPs resulting from the centrifugation, freezing, and lyophilization process is shown. **(A)** presents the results for *S. aureus* and **(B)** for *E. coli.* A stock solution of 80 μg/mL was used for the assay. Then, obtain a concentration gradient from 22 μg/mL to 4 μg/mL.

Figure 8 shows the result of the first approach to determining the MIC of CSNPs after the lyophilization process. The resulting NPs were determined to weigh 100 μg/mL. The lyophilized solution was then resuspended in MiliQ water in the same volume of the synthesis solution (20 mL) to obtain a 5.0 μg/mL concentration solution. This concentration was used to determine the MIC, the results of which are presented in the figure below (Figure 9).

In all the columns exposed to the treatment, the coloration changed from blue to pink, indicative of cell viability (except in the sterility controls). This result indicates that CSNPs at the sampled concentrations were ineffective in adversely affecting the bacteria under study.

Likewise, it was decided to test with higher concentrations of NPs, for which the CSNPs resulting from 3 syntheses were concentrated in a centrifuge tube, and the same lyophilization process was performed. A total weight of 400 μg was determined and resuspended in 5 mL of MilliQ water to obtain a concentration for the stock solution to be used in the MIC tests of 80 μg/mL (Figure 9).

The figure presented above reveals a pink coloration in all columns subjected to the concentration gradient of lyophilized chitosan nanoparticles (CSNPs), except for sterility control columns. This result indicates no adverse effect on the bacteria under study, even at NP concentrations approximately 12 times higher than those assessed in Figure 8. Conversely, the color change from resazurin (blue) to resorufin (pink) is indicative of metabolic activity, i.e., the presence of living cells (Wong et al., 2022; Riss et al., 2016), as shown in Figure 7.

**FIGURE 10:**
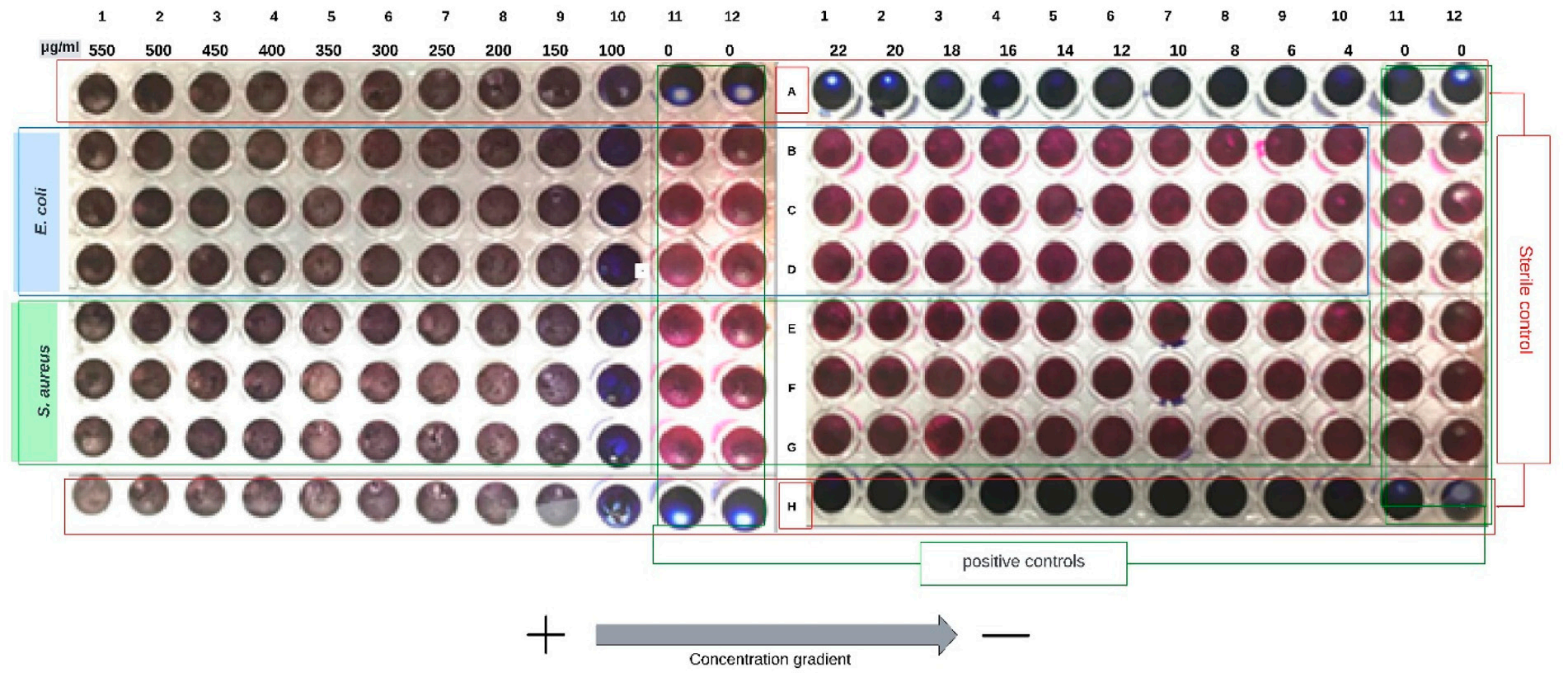
The result obtained for MIC of two initial CS concentrations of 2 mg/mL **(A)** and 80 μg/mL **(B)** is shown. Thus, a first concentration gradient from 500 μg/mL to 100 μg/mL and another from 22 μg/mL to 4 μg/mL is obtained.

Likewise, this analysis was also performed for the mother solution of CS at a concentration of 2 mg/mL and another at 80 μg/mL to determine its antimicrobial effect; the results are shown in Figure 10.

As shown in 10A, all columns are blue, indicating that no cellular metabolism reduced resazurin to resorufin (except in growth control). On the other hand, in 10B, the opposite is presented, where all the columns subjected to the treatment present a pink coloration, indicating a reduction of resazurin to resorufin due to the action of the metabolism of the bacteria under study. With this result, it is possible to have an approximation to the possible minimum inhibitory concentration of CS; it is suggested that the MIC of CS should be between the range of 100 μg/mL and 22 μg/mL, which are the lower and upper extremes, respectively, of both analyses presented in Figure 10. Further analysis within this range of concentrations is required to determine the precise value of the MIC for the CS.

In the study, several tests were performed using the Minimum Inhibitory Concentration (MIC) assay for different treatments, including chitosan nanoparticles (CSNPs) in synthesis solution, lyophilized CSNPs at concentrations of 5 μg/mL and 80 μg/mL, and chitosan (CS) dissolved in 1% acetic acid at concentrations of 2 mg/mL and 80 μg/mL. The results showed inhibition of bacterial growth for CSNPs in the synthesis solution. However, no growth inhibition was observed in any CSNP treatments after centrifugation, freezing, and lyophilization. On the other hand, a possible MIC value was identified for CS dissolved in 1% acetic acid, ranging from 100 to 22 μg/mL.

In the first trial presented in Figure 7, there is an approximate 17% difference in the concentration gradient between column 7 and column 6, suggesting a more pronounced inhibitory effect in *Staphylococcus aureus* than in *Escherichia coli.* However, this result contrasts with the findings of Qi et al. (2004), who reported an MIC value of 0.0625 μg/mL against *E. coli* and 0.125 μg/mL against *S. aureus* for chitosan nanoparticles (CSNPs). The difference between these values is 50%, compared to the 17% observed in this study. Qi et al. (2004) also reported a MIC value of 8 μg/mL for CS in solution, implying a higher effectiveness of NPs compared to dissolved CS; this value is considerably lower than the range of 1,000 μg/mL to 22 μg/mL found in this study for CS. Therefore, a need for much lower concentrations of CSNPs could be expected to achieve inhibition, compared to concentrations of CS in solution. These results align with Katas et al. (2011) findings, who found that NPs had a more significant effect on growth inhibition than CS. Despite this, in the concentration ranges of CSNPs from 1.38 μg/mL to 0.25 μg/mL (Figure 8) and 22 μg/mL to 4 μg/mL (Figure 9), growth inhibition was not achieved for either of the two model bacteria.

As anticipated, chitosan (CS) at higher concentrations showed an inhibitory effect on the growth of *Staphylococcus aureus* and *Escherichia coli* in the range of 500 μg/mL to 100 μg/mL. However, using a CS concentration equivalent to that of lyophilized chitosan nanoparticles (CSNPs) (80 μg/mL) in Minimum Inhibitory Concentration (MIC) assays, bacterial growth was not inhibited. Therefore, it is suggested that the MIC value of the CS should be between the lower (1,000 μg/mL) and upper (22 μg/mL) limits of the concentration gradients evaluated. The results obtained for CS are in line with the MIC values reported by Liu et al. (2004), which indicate MIC values of 20 μg/mL for *E. coli* and *S. aureus*, and with the findings of Devlieghere et al. (2004) and Tsai et al. (2004), who reported MIC values of 100 μg/mL for *S. aureus* and *E. coli,* respectively. Notably, MIC values can vary significantly due to differences in the molecular mass and degree of deacetylation (DDA) of CS, with some studies reporting MIC values as low as 1 mg/mL (Wang et al., 2011). In addition, Shanmugam et al. (2016) reported a MIC value of 80 μg/mL for both bacteria.

Two hypotheses are proposed to explain why it was not possible to determine the Minimum Inhibitory Concentration (MIC) for lyophilized chitosan nanoparticles (CSNPs). The first hypothesis suggests that an adequate concentration of NPs was not reached to inhibit bacterial growth effectively. Therefore, it is proposed that the MIC value should be greater than 22 μg/mL, which was the highest limit of the concentration gradient achieved in the standardized MIC test. To verify this hypothesis, it would be necessary to concentrate the NPs further, resulting from more than three syntheses, and to carry out a new evaluation.

In addition, the impact of filtration on the concentration of NPs must be considered. Before determining the MIC, all samples were filtered through 0.45 μm and 0.22 μm filters to ensure sterility before the assays were used. Käuper and Forrest (2006) reported that filtration of a CSNP solution can reduce its relative concentration by up to 35%. This reduction could vary given that, in their study, 85% of the CSNPs were smaller than 450 nm. In contrast, the CSNPs showed an Average Hydrodynamic Diameter (DHP) of approximately 194 nm in the present study. Therefore, the actual concentration of the NPs used in the post-filtration assays could be significantly lower than that determined by the weight difference after the freeze-drying process.

Another aspect to consider in determining the Minimum Inhibitory Concentration (MIC) of lyophilized chitosan nanoparticles (CSNPs) is that the lyophilization process may have had adverse effects on the stability of the CSNPs. During freeze-drying, CSNPs were centrifuged at 14,000 rpm for 45 min. It has been reported that centrifugation can cause an increase in the size of NPs, probably due to agglomeration or melting processes between particles. Katas et al. (2011) observed an increase in CSNP size from (154 ± 2) nm to (390 ± 7) nm and a reduction in Zeta potential from (47 ± 2) mV to (36 ± 1) mV. It is known that a larger size of NPs can decrease their antimicrobial effect, mainly by reducing the surface area available to interact with bacterial walls. In addition, a decrease in the positive charge of NPs negatively affects this interaction, reducing the antimicrobial efficacy of the system (Suri et al., 2007; Sadeghi et al., 2008; Mohammadi et al., 2016).

Regarding the freezing process, López-León et al. (2005) reported that for CSNPs obtained by ionotropic gelation with tripolyphosphate (TPP), freezing and thawing at −10°C caused a complete destabilization of the system, making it useless for any application. It has been suggested that water crystals formed at freezing temperatures can exert mechanical forces on NPs, which is one of the possible causes of the destabilization of the system (Cesàro et al., 2008).

This study suggests that the lack of inhibition observed in lyophilized chitosan nanoparticles (CSNPs) may be related to adverse effects of the lyophilization process, such as aggregation and decreased surface load, as indicated in Table 6. It was found that the use of cryoprotectants during freeze-drying can mitigate these adverse effects. Abbas (2010) reports that cryoprotectants such as sucrose, mannitol, and sorbitol help preserve the properties of the hydrogel, with sucrose being the most effective in preventing aggregation. Rampino et al. (2013) also evaluated the impact of trehalose, mannitol, and PEG on the size variation of NPs, finding that trehalose 5% offered the best results, probably due to its characteristics such as low hygroscopicity and a high glass transition temperature (Tg). Almalik et al. (2017) suggest using 5% mannitol as a cryoprotectant.

It is essential to consider that the antimicrobial activity of CS is due to the protonation of its amino groups in acid solution and that, at higher pH, the load and ability to interact with bacterial walls decreases. Both CS and CSNPs (80 μg/mL) were suspended or diluted in MiliQ water, which could negatively affect surface loads and NP conformation, as these characteristics are pH-dependent. Qi et al. (2004) reported lower antibacterial activity of CSNPs suspended in MiliQ water than those dispersed in acetic acid.

Therefore, it is recommended that future trials be conducted evaluating the use of cryoprotectants during centrifugation, freezing, and freeze-drying processes and re-evaluating minimum inhibitory concentration assays, even at higher concentrations than those already used in this study.

## 4 Conclusion

In this study, it was possible to successfully synthesize chitosan nanoparticles (CSNPs), identifying the optimal CS: TPP ratio of 4:1. This ratio resulted in desirable, reproducible particle sizes with adequate Zeta potentials that ensure the stability of the system and allow for future modifications in the structure of the nanosystem.

Key aspects, such as the interaction between tripolyphosphate phosphate groups (TPP) and CS amino groups, were determined through various characterization methods, as were the sizes, distributions, and morphology of CSNPs. These findings provide an in-depth understanding of the physical and chemical properties of CSNPs.

The study also addressed the inhibition capacity of CSNPs in their synthesis dissolution, revealing a slight difference in inhibition between *Staphylococcus aureus* and *Escherichia coli*, with *S. aureus* being slightly more sensitive. However, no significant inhibition was observed after the lyophilization process of CSNPs. This suggests that, without cryopreservation treatment, the Minimum Inhibitory Concentration (MIC) value for CSNPs is not in the range of 0.25 μg/mL to 22 μg/mL. In addition, the range for determining the MIC of the CS was approximated between 100 μg/mL and 22 μg/mL. These findings highlight the study’s achievements and limitations, providing a solid foundation for future research in chitosan nanoparticles and their potential use in biomedical applications.

## Data Availability

The original contributions presented in the study are included in the article/Supplementary Material, further inquiries can be directed to the corresponding author.
